# An Unusual Tachycardia

**Published:** 2004-07-01

**Authors:** Sam Hanon, Michael Shapiro, Paul Schweitzer

**Affiliations:** Division of Cardiology of Beth Israel Medical Center, University Hospital and Manhattan Campus for the Albert Einstein College of Medicine New York, NY

**Keywords:** Atrial tachycardia, P wave, atrial electrogram

## Abstract

The following article presents an unusual case of atrial tachycardia, initially misdiagnosed due to a lack of clear P waves. The diagnosis was eventually confirmed using the atrial electrogram from the patient’s pacemaker.

## Case report

An eighty-four year old Dominican man presented to the Emergency Department complaining of “dizziness”. He had a past medical history significant for aortic stenosis, status post aortic valve replacement, paroxysmal atrial fibrillation and sick sinus syndrome, for which a DDD pacemaker was placed. Two days prior to admission he noted light-headedness, which began while walking.

**Figure 1:**
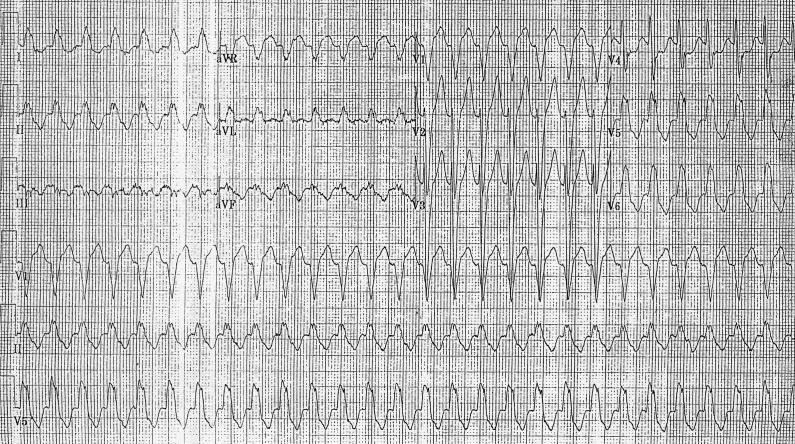
Initial ECG on presentation: Wide complex tachycardia at 170 bpm with LBBB morphology

Initial electrocardiogram (ECG) (Figure 1), revealed a wide complex tachycardia at 170 bpm with LBBB morphology. The differential diagnosis included ventricular tachycardia with exit block, atrial tachycardia, atrial flutter, and A-V nodal reentrant tachycardia. Previous tracings had shown a preexisting LBBB.

**Figure 2:**
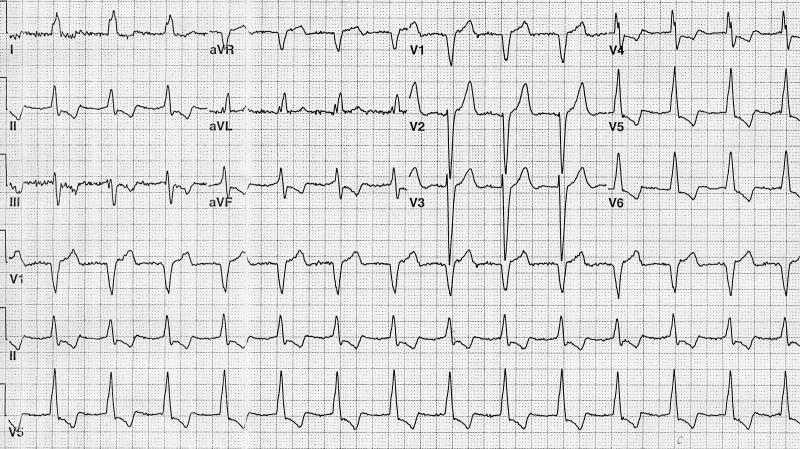
Repeat ECG after resolution of symptoms: LBBB morphology with a ventricular rate of 85 bpm and no clear P wave activity

The tachycardia terminated spontaneously but recurred. After the resolution of symptoms, the ECG was repeated (Figure 2). This ECG showed a QRS morphology similar to the admission tracing with a ventricular rate of 85 bpm and no clear P wave activity.

**Figure 3:**
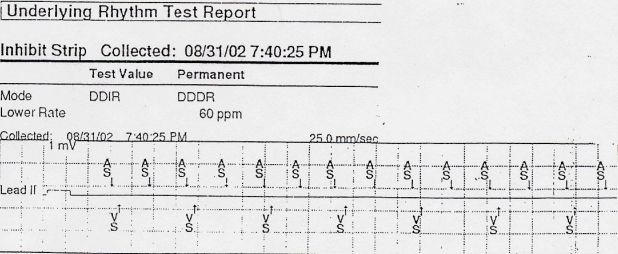
Pacemaker interrogation demonstrating sensed atrial activity at 170 bpm and conduction to the ventricle 2:1 resulting in a ventricular rate of 85 bpm, confirming a diagnosis of atrial tachycardia

Because the follow up ECG was at a rate exactly half the initial one, an underlying atrial tachycardia was suspected. The pacemaker was interrogated to assist in analysis of the rhythm (Figure 3). The interrogation revealed atrial tachycardia with 2:1 block, with an atrial rate of 170 bpm and a ventricular rate of 85 bpm. The patient was treated with metoprolol and his symptoms resolved after normalization of his rhythm.

## Discussion

P wave size and morphology are determined by the location of the ectopic pacemaker and the sequence of atrial activation, which may be influenced by atrial disease [1]. As a result, the correlation between atrial activity and cardiac rhythm can be difficult to establish by surface ECG alone. Specifically, in cases of atrial tachycardia, identification of the underlying rhythm can be problematic, as P waves may be small or hidden in the QRS complex. Occasionally, as in the present case, no P waves may be detected from the ECG while the atrial electrogram demonstrates clear atrial activity. Consequently, the rhythm may be mistaken for junctional tachycardia or A-V nodal reentrant tachycardia. This case illustrates that the atrial wire can facilitate elucidation of atrial activity during an arrhythmia.
